# Identification and Validation in a Novel Quantification System of Ferroptosis Patterns for the Prediction of Prognosis and Immunotherapy Response in Left- and Right-Sided Colon Cancer

**DOI:** 10.3389/fimmu.2022.855849

**Published:** 2022-04-04

**Authors:** Heng-Chun Zhang, Shen-Hui Deng, Ya-Nan Pi, Jun-Nan Guo, Hua Xi, Xin Shi, Xue-Fei Yang, Bo-Miao Zhang, Wei-Nan Xue, Bin-Bin Cui, Yan-Long Liu

**Affiliations:** ^1^ Department of Colorectal Surgery, Harbin Medical University Cancer Hospital, Harbin, China; ^2^ Department of Anesthesiology, The Fourth Affiliated Hospital of Harbin Medical University, Harbin, China; ^3^ Department of Gynecology, Harbin Medical University Cancer Hospital, Harbin, China; ^4^ Department of Rehabilitation Medicine, The Second Affiliated Hospital of Harbin Medical University, Harbin, China; ^5^ The First Department of Oncology, The First Affiliated Hospital of Harbin Medical University, Harbin, China

**Keywords:** colon cancer, left-sided, right-sided, ferroptosis, immunity, prognosis, immunotherapy response

## Abstract

**Background:**

This study aimed to establish a novel quantification system of ferroptosis patterns and comprehensively analyze the relationship between ferroptosis score (FS) and the immune cell infiltration (ICI) characterization, tumor mutation burden (TMB), prognosis, and therapeutic sensitivity in left-sided and right-sided colon cancers (LCCs and RCCs, respectively).

**Methods:**

We comprehensively evaluated the ferroptosis patterns in 444 LCCs and RCCs based on 59 ferroptosis-related genes (FRGs). The FS was constructed to quantify ferroptosis patterns by using principal component analysis algorithms. Next, the prognostic value and therapeutic sensitivities were evaluated using multiple methods. Finally, we performed weighted gene co-expression network analysis (WGCNA) to identify the key FRGs. The IMvigor210 cohort, TCGA-COAD proteomics cohort, and Immunophenoscores were used to verify the predictive abilities of FS and the key FRGs.

**Results:**

Two ferroptosis clusters were determined. Ferroptosis cluster B demonstrated a high degree of congenital ICI and stromal-related signal enrichment with a poor prognosis. The prognosis, response of targeted inhibitors, and immunotherapy were significantly different between high and low FS groups (HSG and LSG, respectively). HSG was characterized by high TMB and microsatellite instability-high subtype with poor prognosis. Meanwhile, LSG was more likely to benefit from immunotherapy. ALOX5 was identified as a key FRG based on FS. Patients with high protein levels of ALOX5 had poorer prognoses.

**Conclusion:**

This work revealed that the evaluation of ferroptosis subtypes will contribute to gaining insight into the heterogeneity in LCCs and RCCs. The quantification for ferroptosis patterns played a non-negligible role in predicting ICI characterization, prognosis, and individualized immunotherapy strategies.

## 1 Introduction

Colon cancer (CC) is a common gastrointestinal malignancy, with an incidence second only to that of gastric and esophageal cancers, and is associated with a high mortality rate (1). Approximately 900,000 people die from CC every year worldwide, accounting for about 10% of the total cancer-related deaths (2). Based on the anatomical structure of the colon, CC can be classified as left-sided CC (LCCs) or right-sided CC (RCCs) (3–5). Previous studies have demonstrated that primary tumor site is an independent prognostic factor in CC, and it is used as a basis for the development of treatment strategies (6–8). Thus, understanding cellular and molecular biological mechanisms between LCCs and RCCs is key in advancing tumor therapy (4).

Multiple studies have identified different CC subtypes based on the tumor cells, immune infiltration, molecular pathways, mutation status, and m6A methylation. Besides, these studies have characterized the differences between these subtypes, including metastasis tendency, recurrence, prognosis, and response to treatment (9–11). Immunotherapy works by recognizing and eliminating tumor cells by activating the body’s natural defense system. Although immunotherapy is gradually becoming the most preferred strategy for cancer treatment, low overall response presents a major hindrance (12). Growing evidence shows that the tumor microenvironment (TME) plays an important role in tumor progression, immune escape, and immunotherapy responses. Recent studies have demonstrated that ferroptosis exerts a dual role in tumor promotion and suppression, as well as affecting the efficacy of chemotherapy, radiotherapy, and immunotherapy. Ferroptosis is an iron-dependent new type of cell death characterized by the accumulation of intracellular reactive oxygen species (ROS). The process is dependent on the release of damage-associated molecular patterns in the TME and the immune response triggered by ferroptosis cell damage. Regulation of ferroptosis can suppress cell migration, invasion, and proliferation of CC (13). On the other hand, β-elemene combined with cetuximab can inhibit the migration of KRAS mutant CC; induce the accumulation of intracellular ROS, glutathione depletion, lipid peroxidation, and transferrin increase; and decrease the negative regulatory protein of ferroptosis, all of which could be reversed by inhibitors of ferroptosis (13). Therefore, targeting the pathway modulating tumor cell ferroptosis is an emerging antitumor strategy, and its combination with other therapeutic approaches could yield huge improvement in the clinical management of cancer. At present, it is possible to use a combination of iron levels, gene expression, and mutations to assess the patients who could benefit from the ferroptosis-promoting treatment. In addition, through a comprehensive analysis of the heterogeneity and complexity of the TME, it is possible to identify different tumor subtypes and novel biomarkers, and the ability to improve prediction of treatment sensitivity. This will also help to find and validate new therapeutic targets. Data on the characteristics of ferroptosis-related subtypes in LCC and RCC remain scanty.

Here, we integrated ferroptosis-related genes (FRGs) and identified ferroptosis-related subtypes in LCCs and RCCs from The Cancer Genome Atlas (TCGA) and Gene Expression Omnibus (GEO) databases. Surprisingly, our data demonstrated that different ferroptosis-related subtypes have unique immune cell infiltration (ICI) characteristics. The data showed that ferroptosis plays a vital role in shaping the TME. Thus, we established a scoring model to quantify the characteristics of ferroptosis and analyzed the relationship between the ferroptosis score (FS) and tumor mutation burden (TMB), prognosis, and treatment sensitivity. Our results provided a novel and accurate method for predicting the prognosis and potential benefits of treatment.

## 2 Materials and Methods

**Figure 1:**
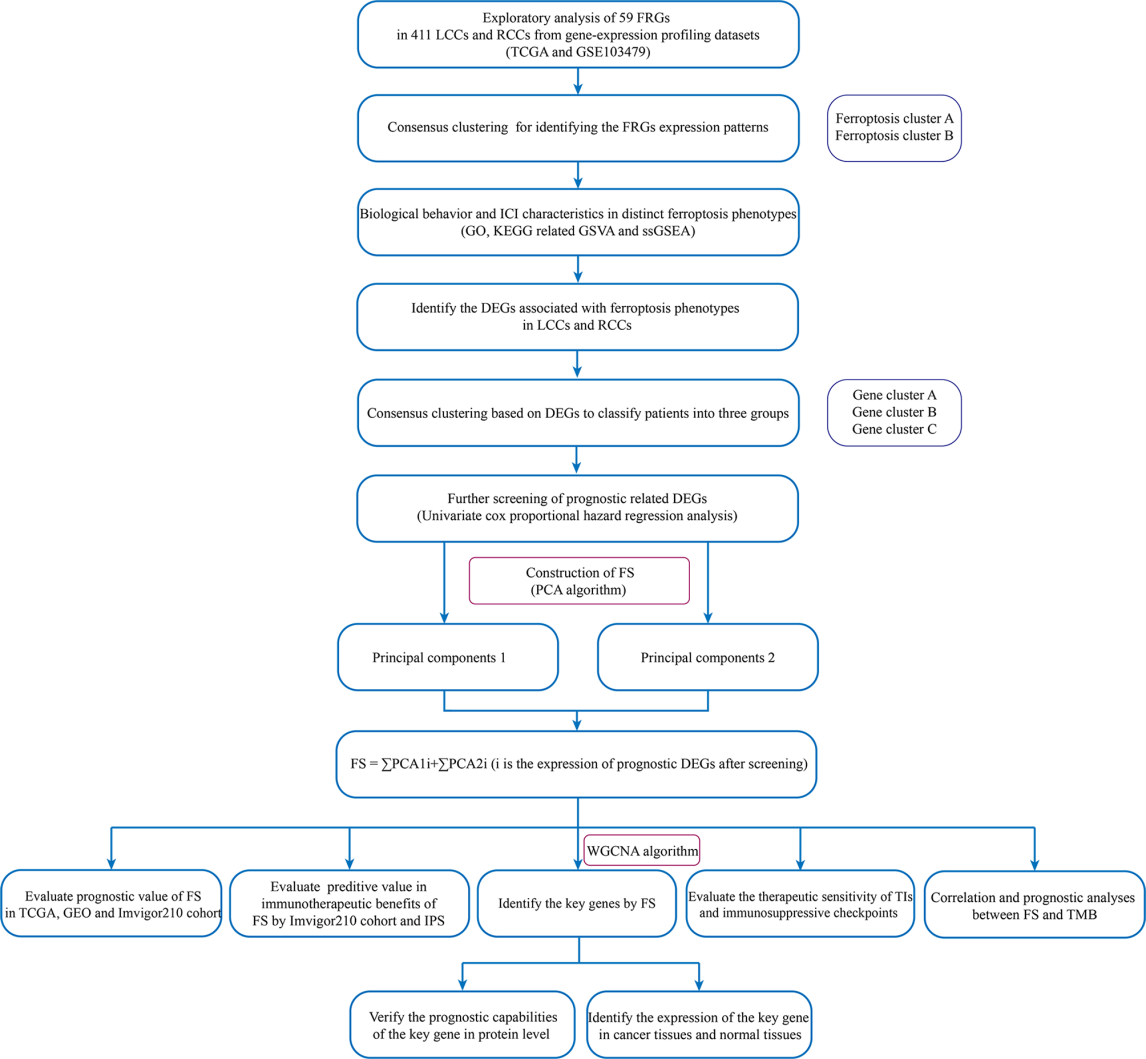
The flowchart of the entire study.

The flowchart of the entire study is shown in **Figure 1**.

### 2.1 Colon Cancer Datasets and Preprocessing

A total of 629 CC samples from two high-throughput platforms were included in this study: 473 samples from TCGA (https://tcga-data.nci.nih.gov/tcga/) and 156 samples from GEO (GSE103479) (http://www.ncbi.nlm.nih.gov/geo/). Other information included somatic mutation information, copy number variation (CNV), primary tumor site, clinical information, and survival data. The inclusion criteria were as follows: 1) the primary sites of the tumor were all in the left or right colon. The tumor primary sites in the cecum, ascending colon, and hepatic flexure are RCCs. The tumor primary sites in splenic flexure, descending colon, sigmoid colon, and rectosigmoid junction are LCCs (2). All patients must have complete follow-up information and RNA-seq data. Patients with incomplete survival information were excluded from the subsequent analysis. Following the screening, 444 samples were admitted to the study, including 322 TCGA samples and 122 GEO samples. The normalized matrix files were downloaded from GEO and RNA sequencing data (FPKM value) of gene expression from TCGA. Then the FPKM value was converted to transcripts per kilobase million (TPM) values for the combined analysis. The “ComBat” algorithm (14) in the R package “SVA” was used to reduce the batch effect caused by non-biotechnology deviations.

### 2.2 Unsupervised Clustering Based on Ferroptosis-Related Genes

To identify different iron ferroptosis-related patterns mediated by FRGs, a total of 59 FRGs was retrieved from previously published literature and available data and extracted the expression of these genes from integrated datasets (15–19). Hierarchical agglomerative clustering was used for sample clustering using the R package “ConsensusClusterPlus” (20). Stability evidence was then employed in unsupervised analysis to determine cluster count and membership. The process was repeated 1,000 times to ensure the stability of clustering.

### 2.3 Gene Set Variation Analysis

To study the differences in biological processes of the ferroptosis subtypes, the R package “GSVA” (21) was used to perform enrichment analysis. The gene set variation analysis (GSVA) was conducted, a non-parametric and unsupervised method, to evaluate enrichment variation, if any, of pathways and biological process activities in different expression datasets. Gene Ontology (GO) and Kyoto Encyclopedia of Genes and Genomes (KEGG) gene sets were downloaded from the MSigDB database (http://software.broadinstitute.org/gsea/msigdb/) and used to perform the GSVA. Heatmaps were used to show the ferroptosis-related pathways with an adjusted *p* < 0.05.

### 2.4 Estimating of Immune Cell Infiltration

To evaluate and quantify the ICI in each sample, single-sample gene-set enrichment analysis (ssGSEA) by the R package “GSVA” (21) was used. After immune cell marker gene expression information was obtained from Charoentong’s research, the enrichment score calculated by ssGSEA was used to represent the relative infiltration abundance of each immune cell. Finally, the differences in ICI between ferroptosis subtypes were analyzed.

### 2.5 Identification of Differentially Expressed Genes Between Ferroptosis Subtypes in Left-Sided Colon Cancer and Right-Sided Colon Cancer

The R package “Limma” (22) was used to identify differentially expressed genes (DEGs) between different ferroptosis subtypes (adjusted *p*-value <0.01). Considering the molecular biological differences, the DEGs in LCCs and RCCs were also identified (|log2foldchange| > 0.5, adjusted *p*-value <0.05). Then the intersection of the two groups of DEGs was taken, and their expression in all samples was extracted for subsequent analysis. The GO and KEGG functional annotations analyses were performed by the R package “clusterProfiler” (23).

### 2.6 Construction of Ferroptosis Score

To quantify the characteristics of ferroptosis in the LCCs and RCCs, an algorithm was developed, and the outcome was defined as FS. First, univariate Cox proportional hazards regression analysis (COX) on the intersection DEGs by the R package “glmnet” (24) was performed. Thereafter, genes with a significant difference in the prognosis were extracted for further analysis. To classify the patients into several groups for in-depth analysis, unsupervised clustering was performed. Afterward, principal component analysis (PCA) was performed to extract the main components of these genes, and then a ferroptosis-relevant gene signature was constructed. Principal components 1 and 2 were selected as signature scores. A method similar to the gene expression rank index was performed to define the FS of each patient: FS = ∑PCA1i + ∑PCA2i (i is the expression of prognostic DEGs after screening). To distinguish the high and low FS groups (HSG and LSG, respectively) associated with prognosis, the best cutoff value was estimated by the R package “maxstat” (25).

### 2.7 Prediction of Multiple Therapeutic Sensitivities

The difference in therapeutic sensitivities between HSG and LSG from targeted inhibitor (TI) therapy and immunotherapy was analyzed. The concentration causing 50% reduction growth (IC50) of TIs was calculated by the R package “pRRophetic” (26), including Notch, Hedgehog (HH), and Wnt inhibitors. Wilcoxon rank-sum test was applied for comparing the difference of IC50 between HSG and LSG.

Meanwhile, immunogenicity is determined by multiple genes, including genes associated with effector cells, MHC molecules, immune regulatory factors, and immunosuppressive cells. Immunogenicity can be estimated and quantified without bias by machine learning. The Immunophenoscores (IPS) of CC were downloaded from The Cancer Immunome Atlas (TCIA) database (https://tcia.at/) (27). Then, the IPS between the HSG and LSG in different immunotherapy methods were compared to predict immunotherapy sensitivity. Meanwhile, a comprehensive search was conducted on the gene expression profile of publicly available datasets treated with immunotherapy, and the metastatic urothelial tumors cohort (28) (IMvigor210: http://research-pub.gene.com/IMvigor210CoreBiologies) and the advanced melanoma cohort (BMS038: https://github.com/riazn/bms038_analysis) (29) were included in the study. The IMvigor210 data were preprocessed by the R package “IMvigor210CoreBiologies.” The RNA-seq data were filtered and normalized by the R package “edgeR” (30), and the data were transformed by “voom” in the R package “limma” (22). The prognostic information and immunotherapy outcomes were also collated. The FS of each sample in the IMvigor210 cohort and BMS038 cohort were also quantified.

### 2.8 Identification of Key Genes Related to Ferroptosis

To identify key prognostic genes related to ferroptosis in the LCC and RCC, weighted gene co-expression network analysis (WGCNA) was performed based on the intersection of DEGs and FS. First, a suitable power exponent was selected to convert the adjacency matrix (AM) to the topological overlap matrix. A correlation analysis between the gene consensus modules with FS was then performed, and the modules negatively correlated with FS were selected for subsequent analyses. The key genes were identified by the intersection of the module genes and the FRGs obtained from the literature. To evaluate the expression of the key FRGs at the protein level, the TCGA-COAD proteomics cohort was downloaded from the Clinical Proteomic Tumor Analysis Consortium (CPTAC) database (https://proteomics.cancer.gov/programs/cptac), which included 29 normal samples and 64 tumor samples. The prognostic value and characteristics of the expression of the key genes were analyzed.

### 2.9 Statistical Analyses

All statistical analyses were conducted by R statistical language (version 4.0.5). Wilcoxon test and Kruskal–Wallis test were used for the comparison between the two groups and more than two groups, respectively. The Kaplan–Meier plotter was used to draw the prognostic survival curve, and the log-rank test was used to evaluate the significance of the statistical difference. Spearman’s test was used for correlation analysis and calculation of correlation coefficient. The R package “maftool” (31) was used to illustrate the gene mutation status in different groups. The R package “rcircos” (32) was used to plot the CNV of FRGs on 23 pairs of chromosomes. In all analyses, *p* < 0.05 was considered statistically significant.

## 3 Results

### 3.1 Genetic Variation of the Ferroptosis-Related Genes in Left-Sided Colon Cancer and Right-Sided Colon Cancer

**Figure 2:**
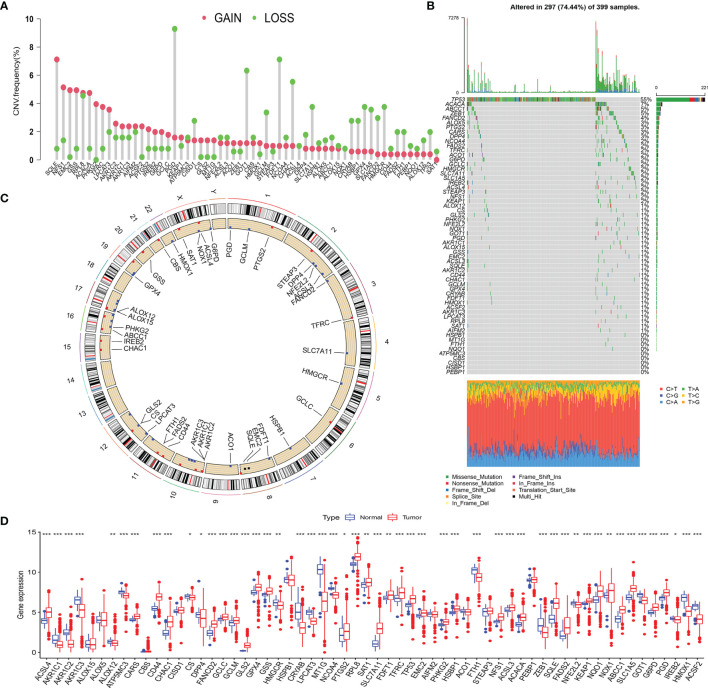
**(A)** The CNV frequency of FRGs in TCGA cohort. The height of the column represents the alteration frequency. The green dot represents deletion frequency. The red dot represents amplification frequency. **(B)** The mutation frequency of 59 FRGs in 399 patients with LCC or RCC from TCGA-COAD cohort. The column represents the patients. The different colors below the figure represent the proportion of variant types. **(C)** The location of CNV alteration of FRGs on 23 chromosomes. **(D)** The difference of FRG expression in normal and tumor tissues. The statistical difference was compared by the Wilcoxon test (^*^ *p* < 0.05, ^**^ *p* < 0.01, ^***^ *p* < 0.001). CNV, copy number variation; FRGs, ferroptosis-related genes; TCGA, The Cancer Genome Atlas; LCC, left-sided colon cancer; RCC, right-sided colon cancer.

Based on recent literature reports, a total of 59 FRGs were included in this study. First, we calculated and demonstrated that FRGs have prevalent CNV alteration in LCC and RCC, and most were focused on the deletion in copy number, while nearly half of the FRGs have a wider frequency of CNV amplification (**Figure 2A**). Out of 399 samples, 297 carried FRG mutations, representing a 74.44% mutation frequency. Among these, TP53 had the highest mutation frequency, followed by ACACA, ABCC1, and ZEB1 (**Figure 2B**). The data showed that there was a frequent occurrence of CNV, and deletions or amplifications of different FRGs had unique characteristics. Genes with a higher frequency of copy number amplification included SQLE, NFS1, EMC2, and GSS. The genes with higher deletion frequency included PGD, GOT1, FDFT1, and CHAC1. We also demonstrated the prevalence of the CNV on these FRGs on the chromosome (**Figure 2C**). To determine whether the above genetic variations affect the mRNA expression in LCC and RCC, we analyzed the mRNA expression levels in normal and cancer tissues and showed that the CNV might be mediating the difference in FRG expression. Compared with normal tissues, the expression of FRGs with higher amplification frequency increased significantly in cancer tissues (e.g., SQLE, NFS1, and ACACA), and vice versa (e.g., GOT1, HMGCR, and FTH1) (**Figure 2D**). In addition, the expression of the FRGs in normal and cancer tissues was highly heterogeneous, indicating that the differential FRG expression plays an important role in the occurrence and development of LCC and RCC.

### 3.2 The Initial Clustering: Ferroptosis Subtypes Have Unique Immune Infiltration Characteristics and Biological Behaviors

#### 3.2.1 Two Ferroptosis Phenotypes Were Identified Based on the Expression Pattern of Ferroptosis-Related Genes

**Figure 3:**
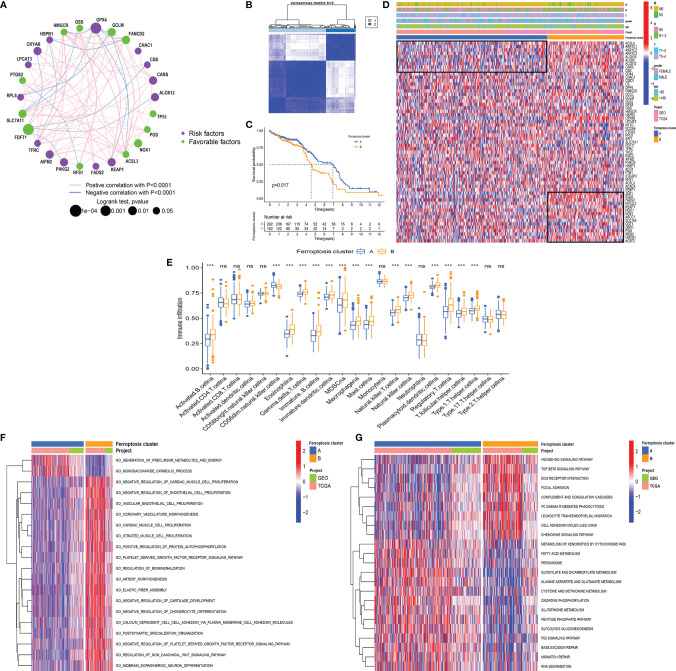
**(A)** The interaction between FRGs in LCCs and RCCs. The circle size represents the range of significance values of each FRG on the prognosis. The *p-*values were calculated by log-rank test. Green dots represent favorable factors for prognosis, and purple dots represent risk factors for prognosis. The lines linking FRGs represent their correlation. The thickness of the lines represents the strength of correlation between FRGs. Negative and positive correlations were marked with blue and red, respectively. **(B)** The consensus matrixes for all CC samples displayed the clustering stability with 1,000 iterations. All samples were clustered into an appropriate number of subtypes (k = 2). **(C)** Kaplan–Meier curves showed the overall survival difference between ferroptosis clusters A and B (*p* = 0.017). **(D)** The heatmap demonstrates the expression of FRGs in different ferroptosis clusters. Heatmap colors indicate relative FRG expression levels. **(E)** The abundance of each ICI in ferroptosis clusters A and B (^***^ *p* < 0.001, ^ns^ *p* > 0.05). **(F, G)** GO-related **(F)** and KEGG-related **(G)** GSVA showing the activation status of biological behaviors in ferroptosis clusters A and **(B)** The heatmap demonstrates these biological pathways. Red and blue represent activated and inhibited pathways, respectively. FRGs, ferroptosis-related genes; LCC, left-sided colon cancer; RCC, right-sided colon cancer; CC, colon cancer; ICI, immune cell infiltration; GO, Gene Ontology; KEGG, Kyoto Encyclopedia of Genes and Genomes; GSVA, gene set variation analysis.

To further explore the interactions, connection, and prognostic impact of the FRGs, we employed univariate COX and correlation analyses on these genes. The results showed that 27 FRGs affected the prognosis (all *p* < 0.05) of the LCC and RCC. There was a significant positive correlation between the FRGs with the same prognostic impact; for example, the KEPA1 expression has a positive correlation with the expression of GPX4, AIFM2, RPL8, and HSPB1 (**Figure 3A**). Meanwhile, there was a significant negative correlation between the prognostic favorable FRGs and unfavorable FRGs, such as the unfavorable factor GPX4 has a negative correlation with favorable prognostic factors HMGCR, GCLM, and FANCD2. The favorable prognostic factor FDFT1 has a negative correlation with unfavorable prognostic factors CRYAB, CBS, and ALOX12 (**Figure 3A**). The data demonstrated that there might be complex crosstalk between the FRGs, which is important for the prognosis of patients and the cell-infiltrating characterization in the TME.

Subsequently, we used the R package “ConsensusClusterPlus” (20) to perform a cluster analysis of the patients based on different FRG expression patterns. The analysis identified 2 ferroptosis-related phenotypes, which were defined as ferroptosis clusters A and B (**Figure 3B**). The prognostic analysis showed that ferroptosis cluster A yielded a better prognosis as compared to ferroptosis cluster B (*p* = 0.017) (**Figure 3C**). Next, we used a heatmap to illustrate the expression pattern of the FRGs and showed that the FRGs were differentially expressed in the 2 clusters (**Figure 3D**).

#### 3.2.2 The Immune Cell Infiltration Characteristics and Biological Behaviors in Distinct Ferroptosis Phenotypes

The analysis of the ICI showed that there was no significant difference in CD4^+^ and CD8^+^ T cells between the two clusters. Although most immune cells in ferroptosis cluster B were highly infiltrated, they contained many immunosuppressive cells, such as eosinophil, myeloid-derived suppressor cells (MDSCs), macrophages, mast cells, and regulatory T cells (Tregs) (**Figure 3E**). These results are somewhat interesting. Previous research has indicated that ferroptotic damage can cause immunosuppression associated with inflammation in tumor ICI (33). Ferroptosis could be induced by cytotoxic T cell-driven immunity (34), but in specific conditions, ferroptosis can promote tumor progression through other immune cells. Ferroptotic cancer cells can release HMGB1 and promote macrophage inflammatory response (35). Additionally, pancreatic cancer cells release KRAS-G12D *via* exosomes during ferroptosis, and macrophages then take up these KRAS-G12D and undergo M2 polarization mediated by AGER to promote tumor progression (36). Neutrophils have been shown to maintain inflammation and promote tumor progression. In lung cancer, neutrophils could secrete pro-inflammatory leukotrienes to change vascular permeability and stimulate cell adhesion (37). The presence of tumor-infiltrating mast cells and FOXP3^+^ Tregs was correlated with the downregulation of HLA-I molecules on tumor cells, resulting in the lack of CD8^+^ T-cell infiltration in these tumor regions (38).

To explore the differences in the biological behaviors between the ferroptosis clusters, we conducted GO- and KEGG-related GSVAs. The results showed that tumor stem cells and stromal-related signals were significantly enriched in ferroptosis cluster B. The signals included the non-canonical Wnt signaling pathway, Hedgehog signaling pathway, TGF β signaling pathway, and extracellular matrix (ECM) receptor interaction (**Figures 3F, G**). The upregulation of the TGF β signaling pathway and ECM receptor interaction reflected the immunosuppressive nature in ferroptosis cluster B. This upregulation pattern was also observed in some stage III colorectal cancer (CRC) regardless of the microsatellite instability (MSI) status (39). Besides, signaling pathways related to genome stability, such as the P53 signaling pathway, base excision repair, and mismatch repair pathway, were significantly enriched in ferroptosis cluster A. The enriched pathways also included ferroptosis-related signaling, such as fatty acid metabolism, peroxisome, glutamate metabolism, and glutathione metabolism. It has been shown that ferroptosis is caused by excessive lipid peroxidation, which can be induced by exogenous or endogenous pathways. The exogenous pathways are initiated by inhibition of cell-membrane transporters, such as cystine/glutamate transporters, while the endogenous pathways are activated by blocking antioxidant enzymes, such as glutathione peroxidase 4 (33). In addition, the p53 signaling pathway is closely associated with ferroptosis and inhibits transcription of SLC7A11, which promotes ferroptosis in cancer cells (40). Some metabolism-related genes, such as SAT1, FDXR, and GLS2, have been reported to be direct targets of p53-mediated ferroptosis under different conditions (41, 42).

### 3.3 The Secondary Clustering: Ferroptosis Phenotype-Related Differentially Expressed Gene Cluster Patients More Stably and Quantify Ferroptosis Patterns to Better Predict Prognosis

#### 3.3.1 The Secondary Clustering Using the Differentially Expressed Genes More Stably Identified Prognostic-Related Subtypes

**Figure 4:**
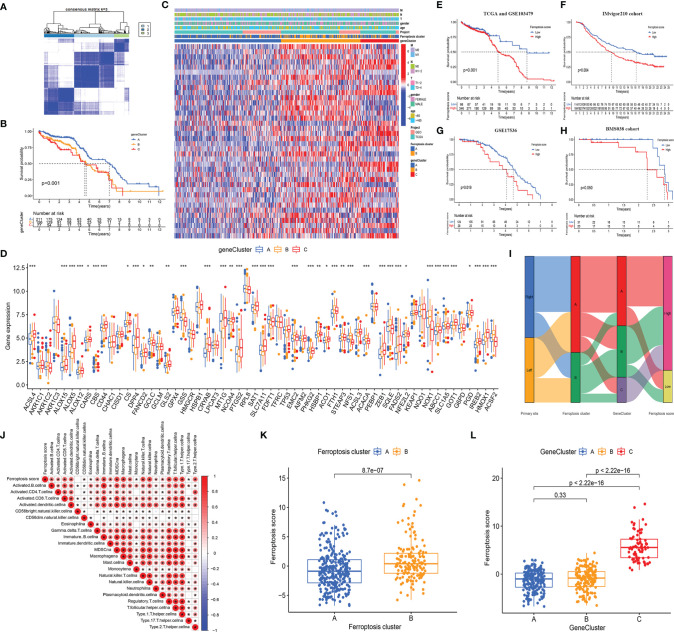
**(A)** The consensus matrixes for TCGA-COAD cohorts based on the DEGs among the 2 ferroptosis clusters. TCGA samples were clustered into an appropriate number of subtypes (k = 3). **(B)** Kaplan–Meier curves showed an overall survival difference between gene clusters (*p* < 0.001). **(C)** The heatmap shows the expression of the ferroptosis-related DEGs in different ferroptosis clusters and gene clusters. **(D)** The difference of FRG expression in normal and tumor tissues. The statistical difference was compared by the Kruskal–Wallis test (^*^ *p* < 0.05, ^**^ *p* < 0.01, ^***^ *p* < 0.001). **(E–H)** Kaplan–Meier curves show overall survival difference between HSG and LSG in TCGA and GSE103479 cohort (*p* < 0.001) **(E)**, IMvigor210 cohort (*p* = 0.004) **(F)**, GSE17536 cohort (*p* = 0.019) **(G)**, and BMS038 cohort (*p* = 0.050) **(H)**. **(I)** The Sankey diagram demonstrates the distribution of patients with primary tumor sites, ferroptosis clusters, gene clusters, and FS. **(J)** Correlations between FS and the abundance of each ICI in TCGA cohort using Spearman’s analysis. Positive correlation with red and negative correlation is marked in blue. **(K)** Differences in FS between 2 ferroptosis clusters. The Wilcoxon test was used to compare the statistical difference between 2 ferroptosis clusters (*p* < 0.001). **(L)** Differences in FS among 3 gene clusters. The Kruskal–Wallis test was used to compare the statistical difference between 3 gene clusters. DEGs, differentially expressed genes; TCGA, The Cancer Genome Atlas; FRG, ferroptosis-related gene; HSG, high ferroptosis score group; LSG, low ferroptosis score group; FS, ferroptosis score; ICI, immune cell infiltration.

To further study the potential biological behavior of each ferroptosis phenotype, we obtained 8,087 DEGs related to ferroptosis phenotypes using the R package “Limma.” In view of the obvious differences between LCCs and RCCs, we analyzed the DEGs in the LCCs and RCCs. After intersection analysis, 508 overlapped DEGs were obtained. GO and KEGG enrichment analyses of the DEGs showed significant enrichment of many biological processes related to immunity, which confirmed that ferroptosis plays a crucial role in TME. To further verify this effect, we performed an unsupervised cluster analysis based on the 508 overlapped DEGs and stratified patients into three ferroptosis phenotype-related DEG clusters (gene clusters A–C) (**Figure 4A**). The stratification aimed at further distinguishing other phenotypic differences caused by the ferroptosis patterns. Under this clustering algorithm, we demonstrated that patients in gene cluster A had the most favorable prognosis (*p* < 0.001) (**Figure 4B**). Most patients in gene cluster A belonged to ferroptosis cluster A, while those in gene clusters B and C with relatively poor prognosis belonged to ferroptosis cluster B (**Figure 4C**). The significant differences in the expression of the FRGs were found within the different gene clusters (**Figure 4D**), which were in accordance with the expected results caused by ferroptosis patterns.

#### 3.3.2 The Ferroptosis Score Had Satisfactory and Consistent Predictive Power for Prognosis in Multiple Independent Cohorts

Given the heterogeneity and complexity of individual ferroptosis patterns and the subsequent identification of key FRGs, we used PCA to quantify the ferroptosis patterns in the LCCs and RCCs and defined the results as FS. Next, we evaluated the value of the FS in predicting prognosis. After the best cutoff value was obtained through the R package “maxstat,” we distributed all the patients from TCGA and GSE103479 cohorts into HSG and LSG. The patients in the LSG showed a better prognosis compared to those in the HSG (*p* < 0.001) (**Figure 4E**). To evaluate the predictive prognostic consistency of the FS, we calculated the FS and performed a prognostic analysis in the Imvigor210 cohort, GSE17536 cohort, and BMS038 cohort. The results showed that the patients in LSG had a significantly better prognosis than those in HSG in the Imvigor210 cohort (*p* = 0.004) (**Figure 4F**), GSE17536 cohort (*p* = 0.019) (**Figure 4G**), and BMS038 cohort (*p* = 0.050) (**Figure 4H**). Thus, the consistency of the predictive effectiveness of the FS was demonstrated, indicating that the FS had robust predictive ability in the cross-validation cohort.

A Sankey plot was used to visualize the primary tumor sites, ferroptosis clusters, gene clusters, and FS in each patient (**Figure 4I**). The LCCs and RCCs patients were classified into 2 ferroptosis clusters and then divided into 3 gene clusters. Compared with gene cluster A, which showed better prognoses, the patients in gene cluster C with poor prognoses belonged to the HSG. Similarly, most patients in gene cluster B with poor prognoses belonged to HSG.

Since the overlapped DEGs were significantly enriched in immune-related pathways, we explored the relationship between the FS and ICI and generated a correlation heatmap (**Figure 4J**). The data showed that there was a significant positive correlation between the FS and most infiltrated immune cells.

In addition, we explored the relationship between the FS and the two clustering modes and showed significant differences in the FS among the different clustering modes. The median FS in ferroptosis cluster B was significantly higher as compared to that in ferroptosis cluster A (*p* < 0.001) (**Figure 4K**). The median FS of gene cluster C was significantly higher than that in gene clusters A and B (*p* < 0.001) (**Figure 4L**). Besides, the patients in ferroptosis cluster B, gene cluster C, and HSG had a relatively worse prognosis, thus demonstrating the consistency of the predictive effectiveness. Therefore, the quantified ferroptosis patterns could be used as an indicator to predict prognosis and ICI.

### 3.4 The Ferroptosis Score Had Significant Associations With Tumor Mutation Burden and Genomic Instability

**Figure 5:**
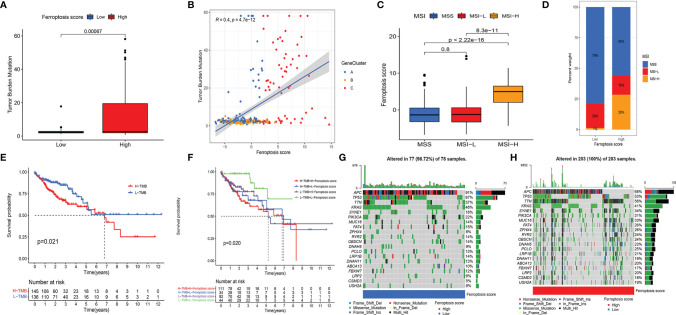
**(A)** Differences in TMB between HSG and LSG. The Wilcoxon test was used to compare the statistical difference between HSG and LSG (*p* < 0.001). **(B)** The scatterplots show that there was a significant positive correlation between FS and TMB. The correlation coefficient between FS and TMB was 0.4 (*p* < 0.001). **(C)** The median FS in the MSI-H subtype was significantly higher than that in MSS and MSI-L subtypes (all *p* < 0.001) **(D)** The proportion of CC molecular subtypes in HSG and LSG. MSS subtype, blue; MSI-L subtype, red; MSI-H, yellow. **(E)** Kaplan–Meier curves show an overall survival difference between TMB subgroups (*p* = 0.021). **(F)** Kaplan–Meier curves show overall survival differences stratified by TMB and FS (*p* = 0.02). **(G, H)** The waterfall diagram demonstrates the top 20 driver genes with the highest mutation frequency in HSG **(G)** and LSG **(H)**. TMB, tumor mutation burden; HSG, high ferroptosis score group; LSG, low ferroptosis score group; FS, ferroptosis score; MSI-H, microsatellite instability-high; MSS, microsatellite stable; MSI-L, microsatellite instability-low; CC, colon cancer.

Our analysis showed that genome stability-related pathways were significantly enriched in the GSVA of ferroptosis clusters. We then performed a series of analyses on somatic mutations in the HSG and LSG. First, we analyzed the difference between the TMB in the HSG and LSG and demonstrated that the TMB in the HSG was significantly higher compared to that in the LSG (*p* < 0.001) (**Figure 5A**). Previous studies have shown that high TMB predicts poor prognosis in a variety of cancers. In our study, the HSG had a worse prognosis, and the results were consistent. Furthermore, there was a positive correlation between the FS and TMB (Spearman’s coefficient: r=0.4, *p* < 0.001) (**Figure 5B**). The distribution of gene clusters varied with the increase of the FS. Gene cluster A with a better prognosis was mainly distributed at the bottom left of the coordinate axis (**Figure 5B**).

CC was classified into microsatellite stable (MSS), MSI-low (MSI-L), and MSI-high (MSI-H) by TCGA project. In the 2017 National Comprehensive Cancer Network guidelines, the immune checkpoint inhibitor anti-PD-1 was recommended as an end-line treatment of CC with deficient mismatch repair (dMMR)/MSI-H subtype [27]. Our analysis showed significant FS differences between the molecular subtypes. The median FS in the MSI-H subtype was significantly higher than that in the other two subtypes (all *p* < 0.001) (**Figure 5C**). A larger portion of patients in the HSG belonged to the MSI-H subtype and had a worse prognosis, while almost all patients in the LSG belonged to the MSS and MSI-L subtypes and had better prognoses (**Figure 5D**). The above results indicated that the ferroptosis patterns not only affect ICI but also have a potential relationship with somatic mutation, which leads to a poorer prognosis of patients under their synergistic influence.

### 3.5 The Synergistic Effect of Ferroptosis Score and Tumor Mutation Burden Further Refined Prognostic Prediction

We performed a prognostic analysis of TMB and found that there was a significant difference in the prognosis of patients in the high and low TMB groups (**Figure 5E**). Taking the synergistic effect of the TMB and FS on the prognosis, we performed a stratified prognostic analysis. We found that patients with a combination of low FS and low TMB showed a great survival advantage (**Figure 5F**). These data indicated that the FS could be a potential prognostic indicator, and the combination with TMB could further refine prognostic prediction for patients.

We then analyzed the differences in the distribution of somatic mutation in the HSG and LSG human populations. The top 20 driver genes with the highest mutation frequency were used for presentation. The data showed that the mutation frequency of the genes in the HSG was generally higher compared to that in the LSG (**Figures 5G, H**).

### 3.6 The Ferroptosis Score Had Great Potential for Predicting Immunotherapy Efficacy

**Figure 6:**
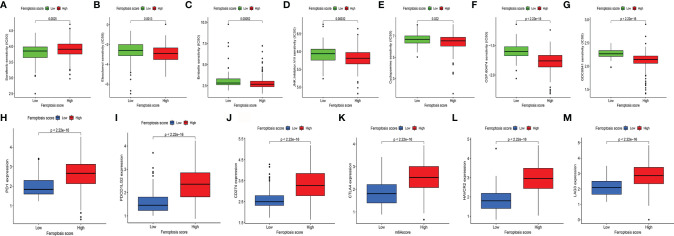
**(A–G)** The difference of multiple TI sensitivity in HSG and LSG. In sunitinib **(A)**, the median IC50 of LSG was significantly lower than that of HSG (*p* < 0.01). In elesclomol **(B)**, embelin **(C)**, JNK Inhibitor VIII **(D)**, cyclopamine **(E)**, CGP.60474 **(F**), and GDC0941**(G)**, HSG had a significantly lower median IC50 than LSG (all *p* < 0.05). **(H–M)** The difference of immune-suppressive checkpoint gene expressions between HSG and LSG. The expressions of PD1 **(H)**, PDCD1LG2 **(I)**, CD274 **(J)**, CTLA4 **(K)**, HAVCR2 **(L)**, and LAG3 **(M)** were higher in HSG than in LSG (all *p* < 0.001). The statistical difference was compared by the Wilcoxon test. TI, targeted inhibitor; HSG, high ferroptosis score group; LSG, low ferroptosis score group.

To evaluate the value of FS in predicting the clinical therapeutic efficacy of CC, we analyzed the difference in sensitivity of TIs between the groups. In sunitinib (VEGFR2 inhibitor) (**Figure 6A**), the median IC50 of LSG was significantly lower than that of HSG (*p* = 0.0025). In elesclomol (inducer of oxidative stress) (**Figure 6B**), embelin (NF-κB inhibitor) (**Figure 6C**), JNK Inhibitor VIII (**Figure 6D**), cyclopamine (HH signaling inhibitor) (**Figure 6E**), CGP.60474 (cyclin-dependent kinase inhibitor) (**Figure 6F**), and GDC0941 (PI3K inhibitor) (**Figure 6G**), the median IC50 of HSG was significantly lower than that of LSG (all *p* < 0.05).

**Figure 7:**
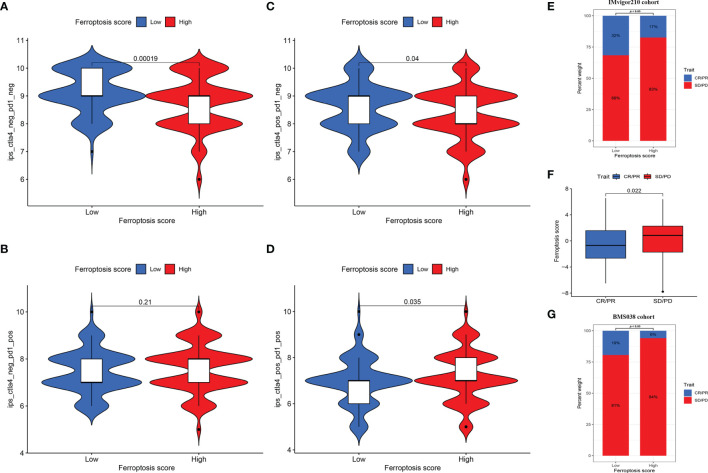
**(A–D)** The differences of IPS between HSG and LSG. The IPS **(A)**, IPS-CTLA4 **(B)**, and IPS-PD1/PD-L1/PD-L2 + CTLA4 **(D)** were significantly different between HSG and LSG (all *p* < 0.05). **(E)** Proportion of patients with different treatment outcomes in HSG and LSG. The proportion of CR/PR patients in LSG was significantly higher than that in HSG in IMvigor210 cohort (*p* < 0.05). **(F)** The difference of FS between treatment outcome groups (*p* = 0.022). The statistical difference above was compared by the Wilcoxon test. **(G)** The proportion of CR/PR patients in LSG was significantly higher than that in HSG in BMS038 cohort (*p* < 0.05). IPS, Immunophenoscores; HSG, high ferroptosis score group; LSG, low ferroptosis score group; CR/PR, complete response/partial response.

For immune-suppressive checkpoint genes, the expressions of PDCD1, PDCD1LG2, CD274, HAVCR2, CTLA4, and LAG3 were higher in HSG than those in LSG (all *p* < 0.001) (**Figures 6H**–**M**). In addition, two methods were performed to verify the predictive ability of FS in immunotherapeutic benefits. Several researchers have identified the ability of IPS calculated by immunogenicity on predicting the immunotherapy response in melanoma patients. So we analyzed the difference of IPS between HSG and LSG. The IPS, IPS-PD1/PD-L1/PD-L2, IPS-CTLA4, and IPS-PD1/PD-L1/PD-L2 + CTLA4 were used to evaluate the potential application of FS. The IPS (**Figure 7A**), IPS-CTLA4 (**Figure 7C**), and IPS-PD1/PD-L1/PD-L2 + CTLA4 (**Figure 7D**) were significantly different in HSG and LSG (all *p* < 0.05). There was no statistically significant difference in IPS-PD1/PD-L1/PD-L2 between HSG and LSG (*p* = 0.21) (**Figure 7B**). The proportion of complete response/partial response (CR/PR) patients in LSG was significantly higher than that in HSG (*p* < 0.05) (**Figure 7E**), and the FS in the CR/PR group was significantly lower than that in the stable disease (SD)/progressive disease (PD) group (*p* = 0.022) (**Figure 7F**). Also, in the BMS038 cohort, the proportion of CR/PR patients in LSG was significantly higher than that in HSG (*p* < 0.05) (**Figure 7G**). Overall, the FS had greater predictive potential in prognosis and immunotherapy efficacy.

### 3.7 ALOX5 Was Identified as the Prognostic Key Gene Based on Ferroptosis Score

**Figure 8:**
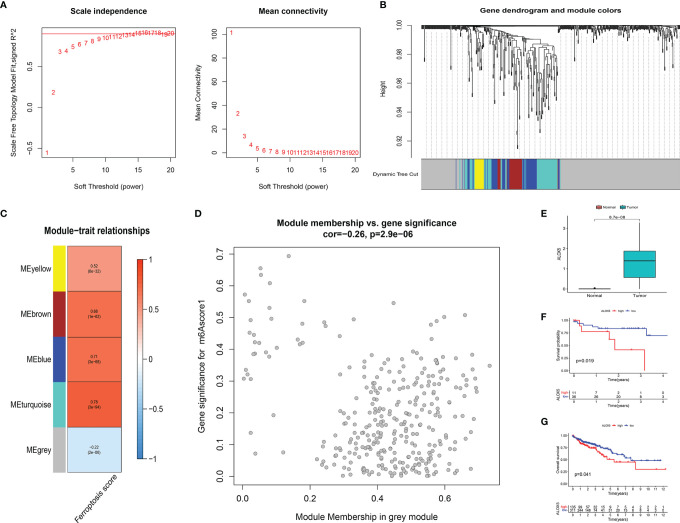
**(A)** The power index 10 was chosen as the appropriate soft threshold, and a scale-free co-expression network was achieved. **(B)** The branches of the dendrogram correspond to 5 gene modules. **(C)** The correlation coefficient and corresponding *p*-value between each gene module and FS are in boxes. **(D)** Scatter plot of module eigengenes in the gray module. **(E)** There were significant differences in ALOX5 expression between cancer tissues and normal tissues at protein level (*p* < 0.001). The statistical difference was compared by the Wilcoxon test. **(F, G)** Kaplan–Meier curves showed overall survival difference between high and low ALOX5 expression groups at protein level (*p* = 0.019) **(F)** and RNA level (*p* = 0.041) **(G)**. FS, ferroptosis score.

To further analyze the key genes, a gene co-expression network was built by using WGCNA to identify modules with the highest correlation with FS. We selected the number 10 as the appropriate soft threshold (**Figure 8A**) and built a scale-free co-expression network. Ultimately, 5 gene modules were obtained (**Figure 8B**). It was apparent that only the gray module had a negative correlation with FS (correlation coefficient = −0.22, *p* < 0.001), while the other modules had a positive correlation with FS (**Figures 8C, D**). To narrow the scope of key genes, we selected the unique gray module for further screening.

To identify the key FRGs associated with prognosis, we selected the genes of the gray module, crossed them with the 59 FRGs collected in the literature, and finally obtained ALOX5. At the protein level, we found that the expression of ALOX5 was significantly more expressed in cancer tissues than in normal tissues (*p* < 0.001) (**Figure 8E**). Meanwhile, patients with higher expression of ALOX5 in protein level and RNA level were all related to poor prognosis (all *p* < 0.05) (**Figures 8F, G**).

Furthermore, the first neighbor of ALOX5 was found in the DisNor database (43) (**Supplementary Figure 1**). The upstream upregulators of ALOX5 included ERK1/2, MAPKAPK2, MAPK1, MAPK3, SP1, and CAMK2A; and upstream downregulators involved zileuton, MBD2, PRKACA, MECP2, and MBD1. Leukotriene A4 was the downstream regulator of ALOX5.

## 4 Discussion

Herein, we first revealed two different ferroptosis clusters using 27 FRGs. The two clusters had significantly different characteristics in the ICI and FRG expression patterns. The analysis of the ICI in the TME showed that ferroptosis cluster A had an immune-desert phenotype, while ferroptosis cluster B had a congenital immune infiltration. The analysis showed that eosinophils, MDSCs, macrophages, mast cells, and Tregs were the main infiltrating immune cells in cluster B. Previous studies have shown that eosinophils have high catalytic content of Fe(II), and increasing the concentration of Fe(II) could yield ROS through Fenton reaction and eventually induce ferroptosis (44). MDSCs have strong immunosuppressive activity, inhibiting the function of T cells and NK cells and promoting immune escape (45). Macrophages can regulate iron metabolism and iron homeostasis (46). The M1 and M2 macrophages formed by macrophage polarization showed iron isolation and iron release phenotypes, respectively (47). Macrophages and iron metabolism play a major role in tumor development. On the other hand, Tregs (Foxp3^+^ CD25^+^ CD4^+^ T cells) have been shown to be recruited by tumor cells in TME to fight antitumor immunity (48). Hong et al. established a ferroptosis-related 12-gene signature in clear cell renal cell carcinoma and demonstrated that macrophages, mast cells, and Tregs were significantly enriched in the FRG model through immunoannotation analysis (49). These results suggested that there was potential regulation between tumor immunity and ferroptosis. In addition, stromal-related signals were significantly enriched in ferroptosis cluster B, especially TGF β signaling pathway and ECM receptor activation, confirming the stromal activation in cluster B. Stromal activation can inhibit antitumor functions of immune cells. Immunosuppressive characteristics might explain the poor prognosis of ferroptosis cluster B. In this study, we obtained ferroptosis-related DEGs and crossed them with the DEGs in the LCCs and RCCs. Enrichment analyses of these overlapped DEGs revealed significant enrichment of many immune-related biological processes, robustly demonstrating that ferroptosis plays a key role in the tumor immune microenvironment (TIME).

Subsequently, we identified 3 ferroptosis-related DEG clusters, which were also significantly associated with stromal and immune activation. We once again demonstrated the importance of changes in the ferroptosis in shaping different TIME landscapes. In addition, we constructed a scoring system to quantify the ferroptosis pattern of each CCs patient, defined as FS, to guide individualized prognostic analysis and precise treatment of CC. Studies have shown that an FRG-based prognostic nomogram could improve the estimation of the survival rate of patients with clear cell renal cell carcinoma (50). Similarly, a novel ferroptosis risk signature could be useful in predicting prognosis and reflecting immune infiltration in adrenocortical carcinoma (51). These analyses also suggested that the FS might be a potential and reliable prognostic biomarker in CC.

Mutations in the DNA damage response genes are the main cause of TMB elevation, which can be used to predict the immune checkpoint inhibitor response (52). Many mutations in the somatic exon region lead to an increase in the production of neoantigens, which are recognized by T cells and thus enhance the antitumor immune response. Therefore, patients with high TMB might develop a stronger immune response and be more sensitive to the immune checkpoint inhibitors treatment. MSI status is used as a symbol of dMMR. dMMR tumors often generate more effective antitumor immune responses and have a higher likelihood of immunotherapeutic responses (53). Le et al. demonstrated that mismatch repair status played a role in predicting the clinical benefit of pembrolizumab immune checkpoint blockade (54). Our data confirmed that the FS could also be predictive indicators for immune checkpoint inhibitor response and prognosis independent of the TMB and is potentially effective in evaluating the MSI status of patients.

Nowadays, there is diversification in cancer treatment strategies. Clinical trial results showed that anti-PD-1 antibodies ultimately achieve a lasting CR in patients with CRC (55). Ferroptosis mediates the tumor-suppressive activity of IFN-γ secreted by CD8^+^ T cells against immune checkpoint blockade. The significant clinical benefits of immunotherapy such as immune checkpoint blockade might be derived, at least in part, from ferroptosis-induced tumor cells. In our analysis, gene expression at immunosuppressive checkpoints was significantly different between the HSG and LSG. Besides, FS can be used to predict the efficacy of TIs and response to immunotherapy. Currently, many clinical trials aim to investigate the use of immunotherapy in combination with a variety of other therapies in the treatment of CC (56–58). Ferroptosis regulators might enable the CC patients to achieve a better therapeutic response by leveraging the iron-philic activity of the immune system in conjunction with immunotherapy and targeted therapy (59, 60).

Given the vital roles played by ferroptosis in TIME and prognosis, we screened out key adverse prognostic regulators. Interestingly, our analysis showed that ALOX5 was a key FRG related to poor prognosis. The main mechanism of ferroptosis is metabolic necrosis caused by the peroxidation of polyunsaturated fatty acids, resulting in the accumulation of toxic products and rapture of the cell membrane (61). ALOX5 is a non-heme iron-containing dioxygenase that encodes lipoxygenase and metabolizes arachidonic acid into 5-hydroperoxyeicosatetraenoic acid (62). ALOX5 was shown to regulate ferroptosis in cancer cells through lipid peroxidation (19, 63). In gastric cancer and hepatocellular carcinoma, ALOX5 expression was significantly higher than that in normal tissues in the promotion of tumor progression (64, 65). On the other hand, ALOX5 inhibition augments the efficacy of other chemotherapeutic agents in the treatment of gastric cancer. Zhou et al. demonstrated that abnormal activation of ALOX5 is associated with HER2 overexpression, mediates the growth and migration of breast cancer, and has prognostic value (66). Previous studies have demonstrated that ALOX5 is associated with macrophage infiltration. ALOX5 induces leukotriene synthesis to create a pro-inflammatory environment. Besides, increased 5-LOX metabolites from hypoxic ovarian cancer cells promote macrophage migration and invasion (67). In this study, we demonstrated the overexpression and adverse prognostic value of ALOX5 in CC. FDA-approved ALOX5 inhibitors already exist. For example, zileuton, a selective ALOX5 inhibitor, is used to prevent and treat chronic asthma (68). In addition, clinical trials have been conducted to evaluate the efficacy of zileuton monotherapy or combination therapy in lung cancer (Clinical trial numbers: NCT00056004 and NCT00070486). We constructed upstream and downstream regulatory networks of ALOX5, whose interaction network could provide the basis for follow-up studies. These results implied that ALOX5 is a potential target for CC tumor therapy and an effective prognostic biomarker.

However, several limitations remain, as follows: we need more independent immunotherapy cohorts to verify the predictive robustness and consistency of FS for prognosis and immunotherapy efficacy. For the key gene, additional experiments are required to investigate the unique role of ALOX5 in LCCs and RCCs.

In summary, this study comprehensively explored the association between ferroptosis and ICI in LCC and RCC. The identification of ferroptosis subtypes will help gain insight into the heterogeneity in LCC and RCC. By establishing a system to quantify ferroptosis patterns, the FS could serve as an effective biomarker to predict prognosis and immunotherapy response. The key gene ALOX5 identified by the FS also showed good predictive abilities. Our study provided a novel tool for the identification of ferroptosis immunophenotypes, predicting prognosis, and provision of individualized immunotherapy in LCCs and RCCs.

## Data Availability Statement

Publicly available datasets were analyzed in this study. These datasets can be found here: TCGA database (http://cancergenome.nih.gov/), the NCBI Gene Expression Omnibus database (GSE103479) (GSE17536) (https://www.ncbi.nlm.nih.gov/), TCIA database (https://tcia.at/) (27), IMvigor210 cohort (http://research-pub.gene.com/IMvigor210CoreBiologies) (28), BMS038 cohort (https://github.com/riazn/bms038_analysis) (29), DISNOR (https://disnor.uniroma2.it/) (43), The CPTAC database (https://proteomics.cancer.gov/programs/cptac).

## Author Contributions

B-BC and Y-LL designed the study. H-CZ, S-HD, and Y-NP drafted the manuscript and collected, analyzed, and interpreted the data. J-NG, HX, and XS drew the figures. B-BC, Y-LL, B-MZ, and W-NX helped with the final revision of the article. All authors have read and approved the final manuscript.

## Funding

This work was supported by the Nn10 Program of Harbin Medical University Cancer Hospital (Nn102017-02), the Post-doctoral Scientific Research Developmental Fund of Heilongjiang (LBH-Q18085), the Youth Science Foundation of Heilongjiang (JJ2018QN0724), and the Natural Science Foundation of Heilongjiang Province (QC2018111).

## Conflict of Interest

The authors declare that the research was conducted in the absence of any commercial or financial relationships that could be construed as a potential conflict of interest.

## Publisher’s Note

All claims expressed in this article are solely those of the authors and do not necessarily represent those of their affiliated organizations, or those of the publisher, the editors and the reviewers. Any product that may be evaluated in this article, or claim that may be made by its manufacturer, is not guaranteed or endorsed by the publisher.

## Supplementary Material

Supplementary Figure 1 The causal interaction of the key gene ALOX5 in DisNor. The database consisted the direct targets and their intracellular localization of ALOX5.
