# Supplementary figures and images for: Identification and Validation in a Novel Quantification System of Ferroptosis Patterns for the Prediction of Prognosis and Immunotherapy Response in Left- and Right-Sided Colon Cancer
Source: Front Immunol. 2022 Apr 4;13:855849. doi: 10.3389/fimmu.2022.855849 (PMC9014300; doi:10.3389/fimmu.2022.855849)

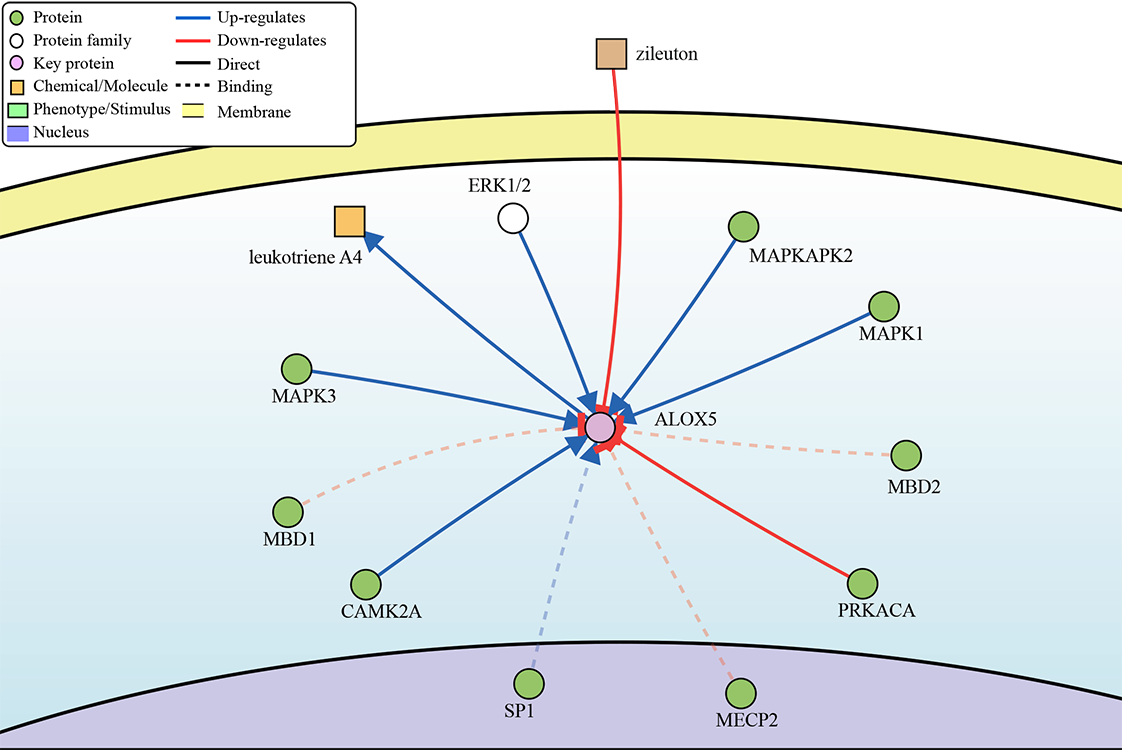

Supplement: Supplementary Figure 1 — The causal interaction of the key gene ALOX5 in DisNor. The database consisted the direct targets and their intracellular localization of ALOX5. [file Image_1.tif]
